# Neck-specific exercise may reduce radiating pain and signs of neurological deficits in chronic whiplash - Analyses of a randomized clinical trial

**DOI:** 10.1038/s41598-018-30556-w

**Published:** 2018-08-17

**Authors:** Maria Landén Ludvigsson, Gunnel Peterson, Anneli Peolsson

**Affiliations:** 1 Department of Medical and Health Sciences, Division of Physiotherapy, Linköping University, Linköping, Sweden; 2 Rehab Väst, County Council of Östergötland, Department of Rehabilitation and Department of Medical and Health Sciences, Linköping University, Linköping, Sweden; 3 Centre for Clinical Research Sörmland, Uppsala University, Uppsala, Sweden

## Abstract

Up to 90% of people with neurological deficits following a whiplash injury do not recover and cervical muscle dysfunction is common. The aim of this multicentre, randomized controlled trial was to examine whether two versions of neck-specific exercise or prescription of physical activity (PPA) can improve radiating arm pain and clinical signs that can be associated with neurological deficits in people with chronic whiplash associated disorders (WAD). Participants with chronic WAD, arm symptoms and signs associated with neurological deficits (n = 171) were randomized to: 12 weeks of neck-specific exercise without (NSE) or with a behavioural approach (NSEB), or PPA. Pain/bothersomeness frequency, six measures of arm pain/paraesthesia (VAS scales), and four clinical neurological tests were evaluated after 3 months. The NSE group reported the lowest frequency and lowest levels of arm pain, the highest proportion of participants with at least 50% pain reduction and the highest proportion of normal arm muscle force. The NSEB group reported increased normal tendon reflexes. No improvements were recorded for the PPA group. Neck-specific exercise may improve arm pain and decrease signs of neurological deficits, but the addition of a behavioural approach does not seem to be of additional benefit.

## Introduction

Up to 90% of people with neurological deficits following a whiplash injury, continue to report symptoms after 1 year^1,2^. Signs associated with neurological deficits in whiplash associated disorders (WAD), may be caused by brachial plexus traction^3^ and/or disc protrusions, which rather seem to progress over time in WAD^4^. Shoulder elevation, as often seen on the painful side in WAD, can reduce brachial nerve tension^5,6^. This may be one reason for the altered muscle function^7^ and lower ability to relax the Trapezius muscle^8^ as detected with electromyography/ultrasound in WAD. Increased activity of superficial muscles may also be a compensatory consequence of the dysfunction and characteristic fatty infiltration of predominantly the deep cervical muscles reported in WAD^9–11^. Since ligaments reportedly account for only 25% of the cervical stability^12^, the deep muscles have an important task maintaining the vertebrae in the positions where loading may be optimally distributed to all supporting structures^13^.

The Quebec Task Force (QTF) classification^14^ of WAD grades is the gold standard used to describe injury sequelae and symptoms; WAD grade 1 = neck complaint without physical signs, 2 = local musculoskeletal signs, 3 = local + neurological signs, including decreased tendon reflexes, muscle weakness and sensory deficits. The classification of grade 3 however offers a clinical challenge, since the number of neurological signs needed is interpreted differently and some of these tests may not be very reliable or sensitive^15^. Other clinical tests, not mentioned by the QTF, such as neural tension tests may be more reliable and sensitive^15^. Furthermore, neither pain^16^, nor muscle weakness, nor abnormal tendon reflexes may be present in all people with cervical radiculopathy^17,18^. Furthermore sensory tests are imprecise considering the overlap of cervical nerve roots. In the absence of universally accepted criteria for the diagnosis of cervical radiculopathy^15^, radiating arm pain and signs of neurological deficits may also be present in other grades. However they are rarely considered in the literature.

People with WAD grade 3 seem to suffer more than those with lower grades^1,19^ and radiating pain is difficult to treat successfully with conventional analgesics^20^. Yet treatment studies rarely include people with WAD grade 3. In a randomized study with chronic WAD grade 2 and 3 from our group, neck-specific exercise, with or without a behavioural approach, focusing on the stabilizing deep muscles, appears to reduce disability^21–23^, improve neck muscle endurance^24^ and psychological variables^25,26^ more than physical activity prescription. Neck-specific exercise was also the cost-effective option^27^. Furthermore treatment success was associated with grade 3 rather than grade 2^28^. However whether arm pain and clinical signs associated with neurological deficits can be improved remains unknown and has, to our knowledge, not been tested before in WAD. We hypothesized that neck-specific exercise would have a positive effect on these outcomes. The aim of this analysis was to examine whether two versions of neck-specific exercise or prescription of physical activity can affect radiating arm pain and clinical signs, which can be associated with neurological deficits in people with chronic WAD.

## Methods

### Design and Procedure

This is a secondary analyses of participants with arm symptoms from a multicentre randomized clinical trial, with assessor and group allocation blinding. Participants in the original study were recruited in 2011–2012^21^. Informed consent was collected before randomization, which was made from a computer-generated list handled by an independent researcher who put the results in opaque envelopes for further distribution to the treating physiotherapists. The study, conducted in accordance with the Declaration of Helsinki, was approved by the Regional Ethics Committee of Linköping University, Sweden.

### Participants

**Figure 1:**
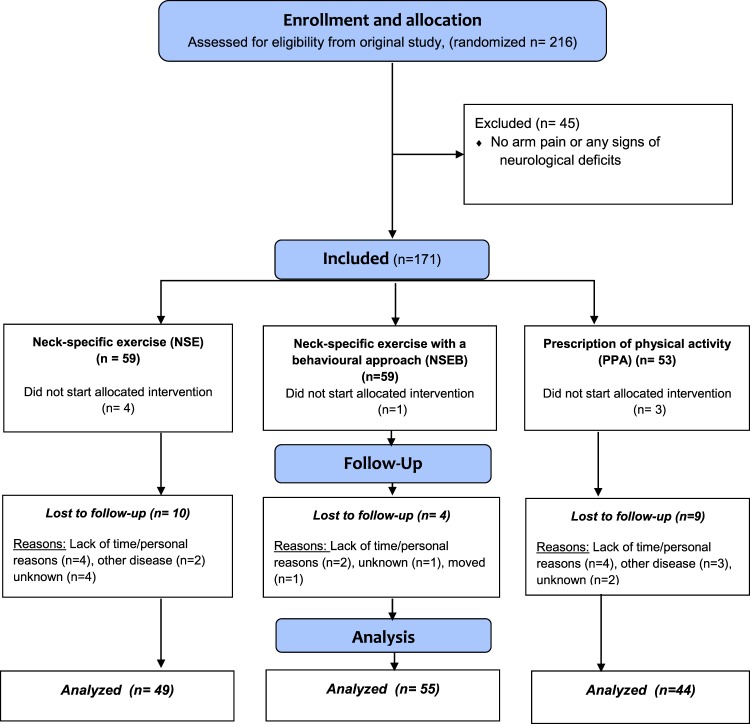
Flow chart of participants.

In the original study, 216 participants with chronic WAD grade 2 or 3 for a duration of 6–36 months were included. From the original study sample, 171 participants with arm symptoms without other known causes and alterations in either sensibility and/or muscle strength and/or reflexes were identified and constitute this study sample (Fig. 1). Additional inclusion criteria for the main study were a Neck Disability Index score (NDI)^29^ of >10/50 points, and/or an average neck pain intensity over the past week on the visual analogue scale (VAS) of >20/100 mm. Exclusion criteria included: previous neck trauma with unresolved symptoms, more dominant pain elsewhere, conditions that were potentially detrimental to completing the study interventions or insufficient knowledge of the Swedish language^21^.

Ninety-one participants (53%) had been categorized as WAD grade 3. The remaining 80 participants included in this analysis (47%, categorized as grade 2) also had arm symptoms without other known causes and at least one of the signs that can be associated with neurological deficits, but this was not enough to classify them as grade 3. The grade 3 classification, was met if two or more of the neurological tests in the physical examination rendered positive observations in the same dermatome/myotome: sensibility, muscle strength, reflexes, and provocation or relief of current arm pain by Spurling’s test of compression or neck traction in lying. This classification has previously been used and found reliable in classification of neurogenic pain^30^. If arm pain was present at the time of the test, a positive neck traction test was mandatory. There were 112 (65%) women and 59 (35%) men with a mean age of 40 (SD 11, range 18–63,) years (Table 1).

**Table 1:** Baseline variables.

	NSE (n = 59)	NSEB (n = 59)	PPA (n = 53)	P
Age, mean (SD)	38 (11)	41 (12)	42 (11)	0.10
*WAD grade 2*	37 (11)	39 (11)	40 (13)	0.67
*WAD grade 3*	39 (11)	42 (12)	45 (9)	0.13
Gender, female, % (n)	71 (42)	69 (41)	55 (29)	0.14
*WAD grade 2*	69 (22)	59 (13)	39 (10)	0.07
*WAD grade 3*	72 (20)	76 (28)	70 (19)	0.89
Neck Disability Index, mean (SD)	17 (6)	18 (7)	18 (7)	0.49
*WAD grade 2*	15 (6)	19 (7)	16 (6)	0.10
*WAD grade 3*	18 (1)	17 (1)	20 (8)	0.34
Months since injury, mean(SD)	19 (8)	20 (9)	20 (11)	0.62
*WAD grade 2*	20 (8)	22 (16)	19 (10)	0.30
*WAD grade 3*	17 (9)	23 (8)	21 (8)	0.09
Smoker, yes % (n)	27 (16)	12 (7)	17 (9)	0.11
*WAD grade 2*	19 (6)	23(5)	15 (4)	0.76
*WAD grade 3*	37 (10)	5 (2)	19 (5)	0.01
Use of analgesic drugs yes (%)*	51 (30)	64 (38)	70 (37)	0.10
*WAD grade 2*	50 (16)	59 (13)	56 (15)	0.76
*WAD grade 3*	52 (14)	68 (25)	81 (22)	0.06
Educational level % (n)				0.64
*WAD grade 2*				0.70
*WAD grade 3*				0.11
*Educational level*, *elementary*	7 (4)	8 (5)	11 (6)	
*Educational level*, *high school*	56 (33)	56 (33)	55 (29)	
*Educational level*, *university*	34 (20)	30 (18)	30 (1)6	
*Educational level*, *other*	3 (2)	5 (3)	2 (1)	
Employed % (n)	75 (44)	76 (45)	67 (36)	0.79
*WAD grade 2*	72 (23)	81 (18)	73 (19)	0.68
*WAD grade 3*	78 (21)	73 (27)	63 (17)	0.47
Neck pain VAS, med (IQR)	38 (21–64)	50 (24–68)	53 (25–61)	0.63
*WAD grade 2*	6 (0–29)	3 (1–19)	2 (0–12)	0.52
*WAD grade 3*	17 (2–46)	6 (1–43)	2 (0–12)	0.27
Positive prov. test % (n),	35 (20)	39 (21)	47 (23)	0.45
*WAD grade 2*	6 (2)	0 (0)	8 (2)	0.44
*WAD grade 3*	67 (18)	57 (21)	78 (21)	0.18

NSE = Neck-specific exercise group, NSEB = Neck-specific exercise group with a behavioral approach, PPA Prescription of physical activity group, WAD = whiplash associated disorder, VAS = Visual Analogue Scale, Neck Disability Index 0–50, Prov.test = Positive Spurling’s and/or neck traction test, med = median, IQR = inter quartile range *Analgesics/NSAID/antidepressants/muscle relaxants, and one participant on gabapentin.

P-values represent between-group comparisons, evaluated with 1-way analysis of variance (ANOVA) for normally distributed parametric data or the Kruskal–Wallis test, with the Mann-Whitney’s U test for post-hoc and Bonferroni post-hoc correction. Bivariate outcomes were evaluated with χ^2^ tests.

### Interventions

All three interventions were undertaken during a 12-week period and treating physiotherapists worked in a primary care setting. The physiotherapists were selected and matched to work within their field of interest and knowledge as much as possible, and with few exceptions only saw participants from one of the groups. A 1-day workshop of training was held by the project leaders, and all physiotherapists were provided with standardized oral and written information about their interventions. The timeframe and specific components of the interventions have been previously published^21,23^ but are presented briefly below. Participants were urged to refrain from having any other physical treatments during the 3-month intervention.

#### Neck-specific exercise (NSE)

Neck-specific exercise with focus on the deep cervical muscles was performed with a physiotherapist twice weekly, along with additional home exercises. After initial unresisted activation of the deep muscles, gym exercises without pain provocation were introduced, with progressive head resistance training in a weighted pulley, focusing on good posture and low load endurance. A detailed description of the exercises can be found at the Academic Archive On-line^31^.

#### Neck-specific exercise with a behavioural approach (NSEB)

The exercises were the same as those undertaken by the NSE group, but in accordance with the concept of behavioural graded exercise, participants were encouraged not to focus on temporary increase in neck pain, but rather on success in exercise progression^32^. Provocation of radiating arm pain was however to be avoided. Participants also received behavioural interventions including education and introduction to activities aimed at pain management (e.g. relaxation, breathing exercises) and problem-solving^21^.

#### Prescription of physical activity (PPA)

The PPA served as the control group without neck-specific exercise. Participants were prescribed individually tailored general physical activity, (e.g. gym classes, Nordic walking) to be performed outside the health care system. It was based on medical history, including also current level of physical activity and a short motivational interview^33^. One follow-up visit or phone call was encouraged.

### Outcome measurements

All outcome measures were collected at baseline and at the 3 month follow-up.

#### Arm pain and paraesthesia

The primary outcome, arm pain, was measured as current arm pain and maximum and minimum level of arm pain in the preceding week on a VAS scale (0 = no pain, 100 = worst imaginable pan). The percentage of participants with a pain reduction of at least 50%, indicating substantial improvement, is also reported, as recommended by The Initiative on Methods, Measurement and Pain Assessment in Clinical Trials (IMMPACT)^34^. Minimum arm pain was used to evaluate whether the pain was constant or intermittent since it can be an important prognostic factor for pain relief^35^. Participants were regarded as having pain free intervals when the minimum level was <3 mm^36^. Secondary outcomes were paraesthesia bothersomeness for the preceding 24 hours (VAS, 0 = not bothersome at all, 100 = extremely bothersome), and frequency of arm pain and of paraesthesia, recorded with a five point scale from never to constantly, as previously used in studies of cervical radiculopathy^18^. Participants filled out the questionnaires at home. Exercise adherence was assessed by examining attendance records from the physiotherapists and participant exercise diaries.

#### Clinical outcomes

All clinical tests (secondary outcomes) were performed by blinded physiotherapists with an average of >10 years’ clinical experience, who also first practiced all tests together to ensure consistency.

Sensitivity was tested with a soft brush and a pinprick wheel at the following locations: supraclavicular space (C4), lateral upper arm below the Deltoid (C5), thumb (C6), 3^rd^ digit (C7), and 5^th^ digit (C8). Responses were classified as normal or abnormal (hypo-, hyper- or dysesthesia, or allodynia). Muscle strength of the Deltoid, Biceps, Triceps, wrist extensors, wrist flexors, finger flexors, and finger abductors was classified as normal or decreased, based on a 6 graded scale from no contraction to normal strength. Deep tendon reflexes (Biceps, Brachioradialis, Triceps) were classified as normal or abnormal (hypo- or hyperreflexia, or areflexia)^37^. Neurodynamic testing, evaluating neural pathology by stressing nervous tissues, was made using the Upper Limb Neural Tension Testing (ULNTT) with median nerve bias^38^. All tests were compared with the uninvolved extremity. As a measurement of overall improvement, a reclassification of WAD grade was also made after 3 months, following the same criteria as previously described.

### Statistics

The sample-size calculation was made for the primary outcome in the main study, the NDI (3.5/50, SD7, alpha 5%, power 80%). The analyses were made on an intent-to-treat basis, including all available patients at either time point. Between-group comparisons were evaluated with 1-way analysis of variance (ANOVA) for normally distributed parametric data or the Kruskal–Wallis test for non-parametric data, with the Mann-Whitney’s U test for post-hoc and Bonferroni post-hoc correction. Due to non-normal distributions VAS-scales were treated as non-parametric (Kolmogorov-Smirnov test, p < 0.05). In binary nominal variables, χ^2^ tests were used. For within-group analyses the Wilcoxon’s signed rank test was used, and for dichotomous outcomes the McNemar test was used. For drop-out analyses independent samples T-tests for parametric data, Mann-Whitney’s U test for non-parametric and χ^2^ tests, or Fisher’s exact test as appropriate for binary outcomes were used. For comparisons between WAD grades, the Mann-Whitney’s U test was used or in case of dichotomous outcomes the McNemar was used. The significance level was set at p < 0.05 (post-hoc Bonferroni correction at p < 0.017). SPSS version 23 (SPSS Inc, Chicago, IL, USA) was used for all statistical analyses. The dataset analysed during the current study is available from the corresponding author on reasonable request.

## Results

### Drop-out analysis

The drop-out rate at 3 months was 15% (n = 25, Fig. 1). There was no difference between drop-outs and completers regarding gender, age or any of the primary or secondary outcomes (all p > 0.28).

### Baseline data and comparison of WAD grades

Sensory deficits were most common at the C4 level (64%) and least common at the C7 level (47%). Fifty-four percent of the participants had sensory deficits at more than two levels. Hyposensitivity was twice as common as hypersensitivity. Muscle weakness was most common in wrist extensors (25%) and least common in finger abductors (16%). Each of the three tendon reflexes was abnormal in approximately 20% of the participants.

Comparing WAD grade 2 and 3 at baseline, participants with WAD grade 3 had significantly more current, minimum and maximum arm pain, and a higher frequency of pain (all outcomes p = 0.01), as well as paraesthesia bothersomeness (p = 0.045) and positive provocation tests (p = 0.00). Grade 3 also had lower proportions of participants with normal muscle force and no provocation of the ULTT-A (p = 0.00), whereas the other two clinical tests were border significant (p = 0.07–0.09). Grade 3 also reported more use of analgesics (p = 0.049). There was also a trend for higher neck disability in grade 3 (p = 0.06). Among participants with WAD grade 3, 93% (n = 85) reported sensory disturbance while muscle weakness was present in 69% (n = 63), and abnormal reflexes in 41% (n = 37). In grade 2, sensory disturbance was the most common finding (85% (n = 68)).

There were no differences between allocation groups in any variables at baseline except regarding smoking in grade 3 (Table 1). No serious harms were reported.

### Arm pain and paraesthesia at follow-up and adherence

The NSE group reported the lowest levels of maximum and minimum pain (with the highest level of pain-free participants) and lowest frequencies of arm pain and of paraesthesia at 3 months (Table 2). The post-hoc tests showed that the NSE group reported less maximum pain and lower frequency of paraesthesia than the PPA group (p = 0.01), and also a lower level of maximum (p = 0.01) and minimum (p = 0.00) pain compared to the NSEB group. Minimum arm pain and arm pain frequency were borderline significant between the NSE and PPA groups (p = 0.02). There was no difference between the NSEB and PPA groups.

**Table 2:** Arm pain and neurological deficits at baseline and follow-up.

										Between group	
	NSE			NSEB			PPA			baseline	3 months
	baseline	3 months	p-value	baseline	3 months	p-value	baseline	3 months	p-value	p-value	p-value
Numbers total sample (n grade 2, n grade 3)	n = 59 (n = 27, n = 32)	n = 49 (n = 27, n = 22)		n = 59 (n = 22, n = 37)	n = 55 (n = 19, n = 36)		n = 53 (n = 26, n = 27)	n = 44 (n = 22, n = 22)			
*WAD grade*, *% (n)*			0.00			0.01			0.74	0.17	0.02*
0/1	0 (0)	12 (6)		0 (0)	9 (5)		0 (0)	0 (0)			
2	54 (22)	59 (29)		37 (18)	46 (25)		49 (17)	46 (20)			
3	46 (27)	29 (14)		63 (37)	44 (24)		51 (27)	54 (24)			
***Self-reported***											
Current arm pain VAS, med (IQR)	11 (1–36)	2 (0–17)	0.03	5 (1–23)	5 (1–31)	0.11	7 (1–25)	12 (0–29)	0.48	0.67	0.07
*WAD grade 2*	*6 (0–29)*	*1 (0–4)*	*0*.*04*	*3 (1–19)*	*5 (1–21)*	*0*.*32*	*2 (0–12)*	*0 (0–20)*	*0*.*36*	*0*.*52*	*0*.*05*
*WAD grade 3*	*17 (2–46)*	*12 (0–28)*	*0*.*27*	*6 (1–43)*	*7 (1–41)*	*0*.*22*	*20 (4–49)*	*29 (3–60)*	*0*.*89*	*0*.*27*	*0*.*17*
Arm pain, minimum VAS, med (IQR)	4 (0–18)	1 (0–7)	0.01	3 (1–14)	5 (1–21)	0.30	2 (0–12)	2 (0–30)	0.12	0.57	0.01^†^
*WAD grade 2*	*3 (0–15)*	*1 (0–12)*	*0*.*01*	*3 (1–12)*	*2 (1–10)*	*0*.*98*	*1 (0–5)*	*1 (0–12)*	*0*.*16*	*0*.*17*	*0*.*01*
*WAD grade 3*	*11 (2–22)*	*3 (0–15)*	*0*.*13*	*4 (1–14)*	*6 (1–24)*	*0*.*24*	*7 (0–27)*	*11 (1–43)*	*0*.*56*	*0*.*68*	*0*.*26*
*No arm pain*, *minimum % (n)*^§^	44 (26)	69 (34)	0.00	44 (26)	36 (20)	0.25	52 (27)	52 (23)	1.0	0.67	0.00
Arm pain, maximum, med (IQR)	25 (1–62)	4 (0–35)	0.00	31 (2–60)	28 (2–60)	0.046	15 (1–49)	34 (4–69)	0.13	0.56	0.01^†^
*WAD grade 2*	*16 (0–45)*	*0 (0–23)*	*0*.*03*	*26 (2–54)*	*16 (2–47)*	*0*.*60*	*4 (0–25)*	*26 (0–44)*	*0*.*16*	*0*.*09*	*0*.*02*
*WAD grade 3*	*50 (9–78)*	*20 (2–67)*	*0*.*13*	*31 (3–61)*	*40 (2–68)*	*0*.*07*	*38 (7–66)*	*61 (12–75)*	*0*.*56*	*0*.*28*	*0*.*24*
Arm bothersomness VAS, med (IQR)	17 (0–43)	4 (0–25)	0.11	20 (1–46)	10 (2–46)	0.60	15 (2–42)	21 (2–56)	0.62	0.82	0.11
*WAD grade 2*	*10 (0–36)*	*2 (0–19)*	*0*.*08*	*19 (2–69)*	*13 (2–43)*	*0*.*39*	*4 (0–22)*	*3 (0–26)*	*0*.*71*	*0*.*52*	*0*.*09*
*WAD grade 3*	*30 (2–47)*	*18 (2–44)*	*0*.*70*	*21 (1–45)*	*7 (2–46)*	*0*.*94*	*36 (10–56)*	*35 (14–73)*	*0*.*79*	*0*.*26*	*0*.*03*
Frequency arm pain		0.00			0.86			0.19	0.31	0.05*	
Occasionally or fewer % (n)	65 (36)	82 (40)		76 (42)	73 (39)		79 (41)	69 (29)			
Daily/several times daily % (n)	35 (19)	18 (9)		24 (16)	27 (14)		21 (11)	31 (15)			
*WAD grade 2*			*0*.*05*			*0*.*49*			*0*.*10*	*0*.*33*	*0*.*23*
*Occasionally or fewer % (n)*	*77 (23)*	*86 (24)*		*57 (17)*	*79 (15)*		*50(23)*	*89 (17)*			
*Daily/several times daily % (n)*	*23 (7)*	*14 (4)*		*19 (4)*	*21 (4)*		*11 (3)*	*11 (2)*			
*WAD grade 3*			*0*.*04*			*0*.*42*			*0*.*51*	*0*.*24*	*0*.*23*
*Occasionally or fewer % (n)*	*52 (13)*	*76 (16)*		*73 (25)*	*71 (24)*		*69 (18)*	*52 (12)*			
*Daily/several times daily % (n)*	*48 (12)*	*24 (5)*		*27 (9)*	*29 (10)*		*31 (8)*	*48 (11)*			
Frequency paresthesia		0.08			0.47			0.30	0.85	0.049*	
Occasionally or fewer % (n)	73 (41)	84 (42)		71 (41)	72 (39)		69 (36)	66 (29)			
Daily/several times daily % (n)	27 (15)	16 (8)		29 (17)	28 (15)		31 (16)	22 (15)			
*WAD grade 2*			*0*.*25*			*0*.*21*			*0*.*74*	*0*.*06*	*0*.*13*
*Occasionally or fewer % (n)*	*87 (26)*	*89 (25)*		*68 (15)*	*84 (14)*		*73 (19)*	*86 (18)*			
*Daily/several times daily % (n)*	*13 (4)*	*11 (3)*		*32 (7)*	*16 (3)*		*27 (7)*	*14 (3)*			
*WAD grade 3*			*0*.*22*			*0*.*92*			*0*.*24*	*0*.*27*	*0*.*08*
*Occasionally or fewer % (n)*	*56 (15)*	*77 (17)*		*72 (26)*	*66 (23)*		*65 (17)*	*48 (11)*			
*Daily/several times daily % (n)*	*44 (11)*	*23 (5)*		*28 (10)*	*34 (12)*		*35 (9)*	*52 (12)*			
***Clinical tests***											
Sensibility, normal % (n)	13 (8)	33 (16)	0.04	5 (3)	24 (13)	0.01	17 (6)	25 (11)	0.18	0.27	0.55
*WAD grade 2*	19 (6)	44 (12)	0.07	9 (2)	26 (5)	0.25	12 (3)	24 (5)	0.62	0.52	0.25
*WAD grade 3*	7 (2)	15 (4)	0.62	3 (1)	22 (8)	0.04	11 (3)	26 (6)	0.38	0.40	0.81
Muscle force, normal % (n)	54 (32)	74 (35)	0.02	51 (30)	63 (35)	0.06	38 (20)	34 (18)	1.0	0.19	0.01^‡^
*WAD grade 2*	72 (23)	85 (23)	0.45	68 (15)	86 (13)	1.0	62 (16)	48 (10)	0.02	0.71	0.02
*WAD grade 3*	33 (9)	60 (12)	0.04	41 (15)	65 (22)	0.02	15 (4)	36 (8)	0.12	0.09	0.10
Tendon reflexes, normal % (n)	61 (36)	76 (37)	0.14	63 (37)	82 (45)	0.03	72 (38)	59 (26)	0.39	0.45	0.04
*WAD grade 2*	66 (21)	74 (20)	0.75	73 (16)	84 (16)	0.69	77 (20)	67 (14)	0.38	0.63	0.45
*WAD grade 3*	56 (15)	77 (17)	0.13	57 (21)	81 (29)	0.04	67 (18)	52 (12)	0.22	0.65	0.05
ULTT, non-prov, % (n)	55 (31)	56 (24)	0.26	57 (33)	56 (26)	1.0	43 (23)	37 (15)	0.75	0.28	0.14
*WAD grade 2*	57 (17)	76 (19)	0.23	68 (15)	81 (13)	1.0	69 (18)	61 (11)	0.63	0.56	0.38
*WAD grade 3*	30 (8)	28 (5)	1.0	50 (18)	43 (13)	0.73	19 (5)	18 (4)	1.0	0.03	0.15

NSE = Neck-specific exercise group, NSEB = Neck-specific exercise group with a behavioral approach, PPA Prescription of physical activity group, WAD = whiplash associated disorder, VAS = Visual Analogue Scale, Neck ULNTT = Upper limb neural tension test, non-prov = non-provocative of pain (or *familiar* pain in ULNTT), med = median, IQR = inter quartile range.

*Significant between NSE and PPA only. ^†^Significant between NSE and both NSEB/PPA. ^‡^Significant between both NSE/NSEB and PPA. ^§^No pain, <3 mm on a 100 mm VAS scale, when the level of pain is at its lowest. The between-group p-values were evaluated with the Kruskal–Wallis test, with the Mann-Whitney’s U test for post-hoc and Bonferroni post-hoc correction. Dichotomous outcomes were evaluated with χ^2^ tests (whole group) or Fisher’s exact test (subgroups). For within-group analyses the Wilcoxon’s signed rank test, and for dichotomous outcomes the McNemar test were used.

**Figure 2:**
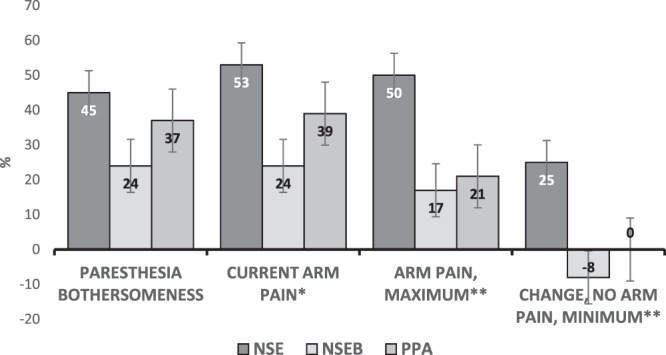
Proportion of participants with 50% reduction in arm pain/paresthesia bothersomeness, and change in proportionof participants with no minimum arm pain. No minimum arm pain <3 mm VAS. NSE = neck-specific exercise, NSEB = Neck-specific exercise with a behavioral approach, PPA = Prescription of Physical Activity *p < 0.05, **p < 0.01. Bars represent standard errors.

Current arm pain (p = 0.07) and paraesthesia bothersomeness (p = 0.11) were not significantly different between groups. However the percentage of participants with at least 50% reduction of current (p = 0.04) and maximum arm pain (p = 0.00), and with pain free intervals (minimal arm pain <3 mm) (p < 0.01) was higher in the NSE group (Fig. 2). The NSE group was also the only group which reported significant within group improvements. When separating the two WAD grades, the NSE group reported significant improvement in current, minimum and maximum arm pain in grade 2, and arm pain frequency in grade 3 after 3 months. The difference between groups was significant in minimum and maximum arm pain grade 2, and bothersomeness in grade 3 (Table 2).

Adherence (at least 50% attendance) was 71% and 69% in the NSE and NSEB group respectively and 45% in the PPA-group (p = 0.05).

### Clinical outcomes at follow-up

After 3 months the two neck-specific exercise groups could be reclassified with lower WAD grades (p NSE = 0.00, NSEB = 0.01) which was not the case for the PPA group (p = 0.74). Nine (NSEB) to 12% (NSE) of the participants were completely free of physical signs of WAD, versus 0% in the PPA group (Table 2).

The percentage of participants with normal muscle force was highest in the NSE group (p = 0.01) and normal tendon reflexes were highest in the NSEB group (p = 0.04). There was no difference between groups regarding sensibility or neural tension (Table 2), even though both neck-specific groups had improved regarding sensibility (p = NSE 0.04, NSEB 0.01). The change in the PPA group was insignificant (p = 0.18).

Separating WAD grades, muscle force was significantly improved in grade 3 (p = NSE 0.04, NSEB 0.02). The only significant change in the PPA group was reduction in muscle force in grade 2 (p = 0.02), which led to a significant difference between groups in grade 2. There were no other significant differences between groups.

## Discussion

### Main findings

The findings in this study suggest that people with persistent arm pain (with a mean duration of almost 2 years) and signs which can be associated with neurological deficits after a whiplash injury, seem to both tolerate and benefit from neck-specific exercise. The NSE group reported the lowest levels of maximum and minimum arm pain (with the highest level of pain-free participants) and lowest frequencies of arm paraesthesia after 3 months. The proportion of participants with at least 50% pain reduction, indicating clinically relevant “substantial improvement” according to IMMPACT^39^, further supports the results of the NSE exercise where this level of improvement was reported in about 50% of the participants. The NSE group was also the only group with an increase of participants (additional 25%) with pain free intervals. To sometimes be pain free as opposed to having constant pain, may allow for temporary recuperation and can be associated with possibilities of further pain relief^35^.

The behavioural approach (NSEB) however rather seems to have counteracted the effect of the neck-specific exercise regarding arm pain. Contrary to the NSE group, temporary neck pain provocation was allowed in the NSEB group, and even though radiating arm pain was to be avoided, the focus was not on pain but on exercise progress, which may have led to participants also ignoring radiating pain.

Regarding clinical signs however, improvements were seen both in the NSE and NSEB groups. Improvements were significant in two out of four outcomes in the NSE group, and in three out of four in the NSEB group and both groups could also be reclassified with lower WAD grades after 3 months as opposed to the PPA group. This implicates that even though the behavioural approach rather seemed to have had a negative impact on arm pain, it was not detrimental to the clinical manifestations. Even though the NSE/NSEB groups improved significantly regarding sensibility by 154% (NSE) and 380% (NSEB), the difference between groups was insignificant since there was also a trend towards improvement by 47% (though not significant) in the PPA group. Even after decompression surgery, sensory deficit tend to improve gradually over time^40^ and further changes may thus occur in a longer perspective. The PPA did not result in significant improvements in any of the outcomes and hence our result do not support the prescription of PPA in this population. Adherence was lower in the PPA group, however excluding those with less than 50% adherence from the analyses did not alter the results. Consistent with the concept, participants in the PPA group only had 1 to 2 physiotherapist visits, whereas the other 2 groups had regular physiotherapist contact which may have influenced the results. Some participants may have felt that the PPA intervention was less specific to their problem and may have been less motivated. However, others may have preferred unspecific approaches in fear of overloading their neck.

### Separate WAD grades

When separating the two WAD grades, the subgroups rendered fewer significant differences between groups (minimum and maximum arm pain and muscle force, grade 2, and bothersomeness in grade 3). This was probably due to the smaller numbers and thus insufficient power, since there was a trend of improvement regarding pain in the NSE group for both grades (though significantly so mostly in grade 2). The median insignificant pain improvements in grade 3 ranged from 29% (current pain) to 73% (minimum pain). The PPA group reported higher or unchanged pain scores but without any significant changes. There was no clear trend in the NSEB group, where there was a mixture of higher and lower scores and no significant changes regarding the pain outcomes.

Regarding clinical outcomes, when analysing the WAD grades separately, there were no significant differences between groups, except in muscle force for grade 2, which can be explained by the deterioration of the PPA group. Just like in the pain outcomes, there was a trend for improvements in the NSE group (significantly so in muscle force, WAD grade 3), but also for the NSEB group (significantly so in WAD grade 3 for all but the ULTT-A). In the PPA group there was a mixture of trends, with significant deterioration of muscle force in grade 2.

### Comparison with other studies

To the best of our knowledge there are no other exercise studies evaluating the effect on arm pain or signs which can be associated with neurological deficits in WAD, but our findings are in line with studies of other neck pain patients with arm pain. A randomized study of women with non-specific neck pain, reports that a multimodal program including neck-specific exercise is significantly better at reducing arm pain, than advice on aerobic exercise and stretching^41^. Another study compared physiotherapy (exercise, including neck-specific exercise, plus a program including pain-coping strategies), with the same program plus surgery in cervical radiculopathy. Both groups improved, and there was no difference between surgery plus physiotherapy or physiotherapy alone regarding arm pain^42^.

### WAD classification and tests

Not all WAD grade 3 participants had positive tests of all three deficits (sensory deficits/muscle weakness/abnormal deep tendon reflexes). The proportion is however similar to what has previously been reported in people with MRI-verified radiculopathy awaiting surgery^18,43^. This further strengthens the interpretation of the QTF classification that not all three tests need to be positive. Furthermore it should be acknowledged that the most common clinical tests of neurological deficits (sensibility, tendon reflexes and muscle force), used in clinical practise all over the world, and upon which the QTF classification is based, are of limited reliability and validity^44,15^. Other tests such as the upper limb tension test A (ULTT-A) and the neck distraction test, reported to have better likelihood ratios^15^, may be useful as a complement. Therefore the results of the clinical tests, both in this study, and in clinical practise, should be interpreted with caution.

There is no evidence to support the use of neither analgesics/non-steroid anti-inflammatory drugs, muscle relaxants nor antidepressants for radicular pain^20^, yet they are often part of the traditional treatment. As opposed to medications, neck-specific exercise is free of side-effects. It may therefore be an important alternative to pain medications. Neck-specific exercise may reduce morphological changes in the deep stabilizing cervical muscles in chronic WAD^45^. However whether the positive effect on arm pain can be explained by an improved ability to maintain the vertebrae in positions where loading is optimally distributed needs to be determined in future studies.

### Limitations

Since this was a secondary analysis, the sample-size calculation for the main study was not based on arm symptoms. Nonetheless significant differences were found in most outcomes, suggesting sufficient power for these outcomes. However the power may have been too low to determine whether any group differences truly exist regarding the insignificant outcomes (less than 49% observed power). This is also even more important to consider in the smaller sub-analyses where WAD grades were separated. Furthermore, other outcomes from the main study have been presented elsewhere, increasing the risk of mass significance. However, since our results are consistent with nearly all previously published outcomes^23–26^ (suggesting a better outcome for the NSE/NSEB groups) it is unlikely that the results in this study can be attributed to chance alone.

Another limitation is that, even though the physiotherapists were matched to work within their field of interest and knowledge, the one day education and the following month with possibilities to practise in their own clinics before seeing any study participants, may have been insufficient to fully master exercises that they were currently not implementing.

Radiculopathy from the levels C4 and above was not evaluated. Difficulties include the lack of specific deficits^17^. Nonetheless it remains an important challenge for future studies to consider, since pain appears more often in the upper part of the cervical spine in chronic WAD than in those with chronic insidious neck pain^46^.

Whether arm pain and deficits found in those with grade 2 were truly neck related nerve pain/deficits cannot be verified. Signs of central sensitization were not further investigated. However hyposensitivity, which is not a feature of central sensitization^47^, was twice as common as hypersensitivity. People with chronic WAD grade 2 and cervical radiculopathy are characterized by a similar upper limb sensory presentation^48^. Furthermore, muscle weakness may have other unknown causes. Nonetheless, muscle force of the upper extremities and most arm pain outcomes did improve following neck-specific exercise without any upper extremity exercise, as opposed to the PPA group, where upper extremity exercises may even have been part of the program. This suggests a neck-related reason, at least to some extent, for their symptoms.

In conclusion this analysis suggest that neck-specific exercise may improve arm pain and decrease signs associated with neurological deficits, but the addition of a behavioural approach does not seem to be of benefit.
